# Correlation between activated clotting time monitoring and heparin concentration measurement in a patient with antiphospholipid syndrome during cardiac valve surgery: a case report

**DOI:** 10.1186/s40981-021-00427-x

**Published:** 2021-03-14

**Authors:** Koichi Yoshinaga, Yuji Otsuka, Taku Furukawa, Shizuka Amitani, Naoyuki Kimura, Masamitsu Sanui

**Affiliations:** 1 Department of Anesthesiology and Critical Care, Jichi Medical University Saitama Medical Center, 1-847, Amanumacho, Omiya-ku, Saitama-city, Saitama, 330-8503 Japan; 2 Department of Cardiovascular Surgery, Jichi Medical University Saitama Medical Center, Saitama, Japan

**Keywords:** Antiphospholipid syndrome, Activated clotting time, Heparin, Cardiopulmonary bypass

## Abstract

**Background:**

Anticoagulation management of patients with antiphospholipid syndrome (APS) undergoing cardiac surgery is challenging due to the prolongation of activated clotting time (ACT). Currently, no study has compared the utility of ACT monitoring using the Hemochron Jr. Signature+ and that of heparin concentration management using the Hemostasis Management System (HMS) Plus in patients with APS.

**Case presentation:**

A 71-year-old woman with APS was scheduled to undergo an aortic valve replacement for aortic regurgitation. The ACT was measured using the Hemochron Jr. Signature+, and the heparin concentration was measured concurrently using the HMS Plus. ACT over 480 s corresponded to an adequate heparin concentration during cardiopulmonary bypass. The clinical course was uneventful, and no thrombotic or hemorrhagic complications were observed.

**Conclusion:**

In the present patient with APS, the Hemochron Jr. Signature+ was useful as an anticoagulation management during cardiac valve surgery.

## Background

Antiphospholipid syndrome (APS) is an acquired hypercoagulable disorder characterized by arterial and/or venous thrombosis and recurrent fetal losses owing to the presence of antiphospholipid antibodies [1]. Patients with APS undergoing cardiac surgery have an increased risk of mortality and morbidity due to perioperative thromboembolic and bleeding events [2, 3]. Difficulty in the intraoperative anticoagulation management of patients with APS is attributed to the spontaneous prolongation of coagulation tests, such as activated partial thromboplastin time (APTT) and activated clotting time (ACT) under the influence of antiphospholipid antibodies. Such prolongation in vitro does not correspond to the in vivo hypercoagulable tendency. Patients who undergo cardiac surgery with cardiopulmonary bypass (CPB) require heparin administration for anticoagulation; however, monitoring the effect of heparin becomes extremely difficult due to the inherent prolongation of ACT [4].

The measurement of whole blood heparin concentration by automated heparin-protamine titration with the Hemostasis Management System (HMS) Plus (Medtronic, Minneapolis, MN, US) is useful for ensuring adequate heparin concentrations during CPB, but its availability is limited due to its complexity. Meanwhile, the discrepancy in ACT measured with the Hemochron Jr. Signature+ and the Hemochron 801 (both devices: Instrumentation Laboratory, Bedford, MA, USA) was previously reported in a patient with APS [5]. The ACT values measured by the Hemochron 801 were out of range during CPB, whereas the ACT values measured by the Hemochron Jr. Signature+ reflected a normal response to heparin. To our knowledge, no study has compared the utility of the Hemochron Jr. Signature+ and that of the HMS Plus in patients with APS. Here, we report a case of successful anticoagulation management in a patient with APS undergoing cardiac surgery, simultaneously using the Hemochron Jr. Signature+ and the HMS Plus.

## Case presentation

A 71-year-old woman presented to our hospital with a diagnosis of severe aortic regurgitation. She had been diagnosed with APS and Sjogren’s syndrome at 35 years of age and had a history of five miscarriages. Preoperative transthoracic echocardiography revealed a left ventricular end-diastolic diameter, left ventricular end-systolic diameter, and left ventricular ejection fraction of 65 mm, 47 mm, and 53%, respectively. Color flow Doppler imaging revealed a central regurgitant jet from the aortic valve. The pressure half-time was 281 ms, indicating moderate aortic regurgitation. Preoperative medication included aspirin as anticoagulation for APS, but not warfarin due to increased risk of bleeding with thrombocytopenia. Prolonged APTT of 101.2 s (reference range, 28.5–40.9 s), normal prothrombin time (PT) of 12.5 s (reference range, 10.3–12.6 s), and marked thrombocytopenia (platelet count, 55 × 10^9^/L [reference range, 130–369 × 10^9^/L]) were observed. Lupus anticoagulant was detected using the dilute Russell’s viper venom time (dRVVT) assay (dRVVT screen ratio 2.08 [reference range, < 1.3]). The patient tested negative for anticardiolipin immunoglobulin G/immunoglobulin M antibodies and anti-β2 glycoprotein I (β2GPI) antibodies. Aortic valve replacement using a bioprosthetic valve was planned.

The results of the baseline ACT varied based on the measurement systems: 101 s, Hemochron Jr. Signature+; 308 s, Hemochron 401; and 136 s, and Hemochron Response (all devices: Instrumentation Laboratory, Bedford, MA, USA). In addition to measuring the ACT with the Hemochron Jr. Signature+, the whole blood heparin concentration was monitored using the HMS Plus. The target heparin concentration was defined as 2.5–3.0 mg/kg (the HMS Plus provides heparin levels in mg/kg: 2.5–3.0 mg/kg is equivalent to 3.4–4.0 U/mL).

**Fig. 1:**
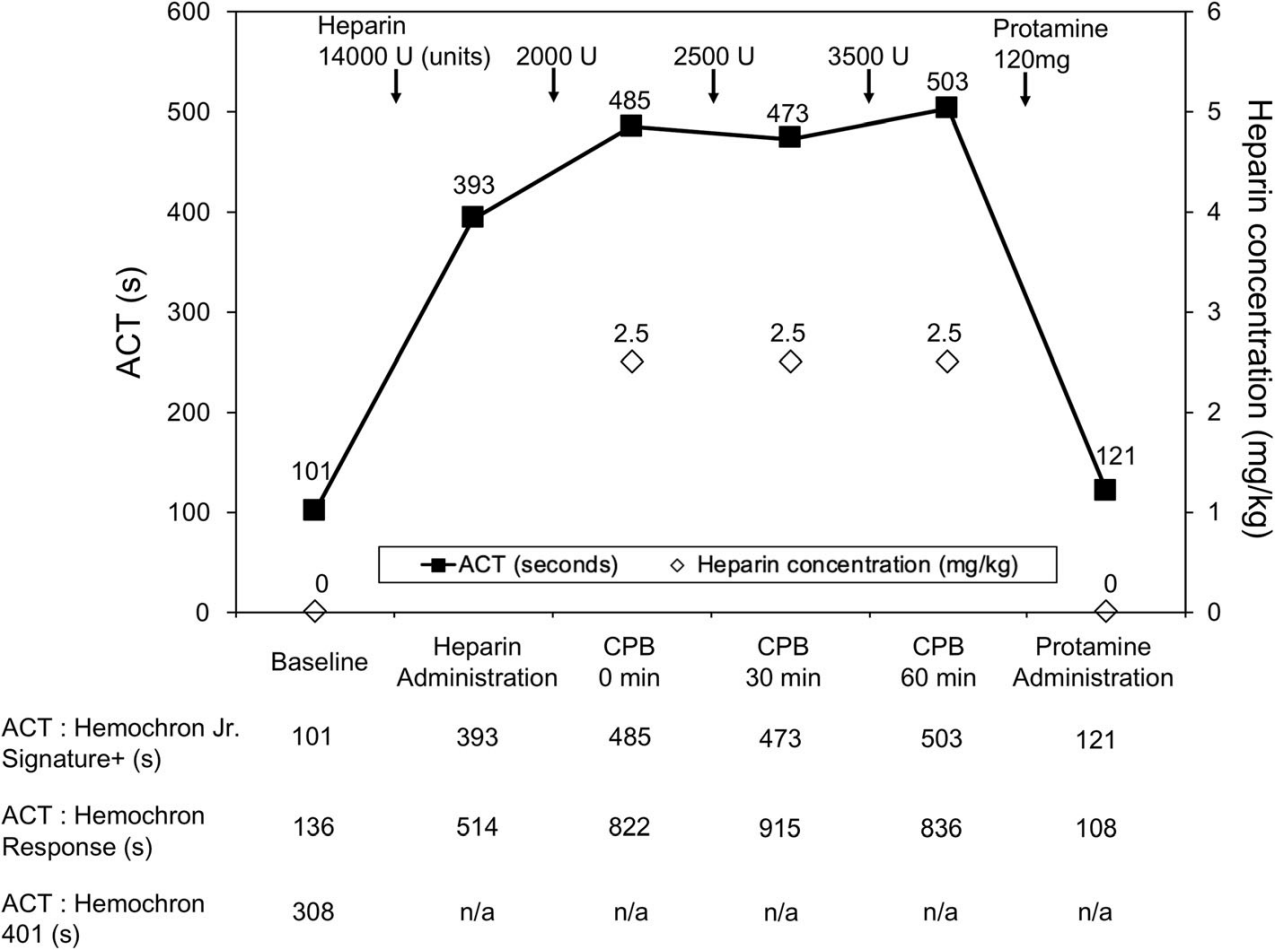
Activated clotting time (ACT) and heparin concentration during cardiopulmonary bypass (CPB). ACT was measured with the Hemochron Jr. Signature+, and heparin concentration was measured by heparin-protamine titration test using Hemostasis Management System Plus. The dose of heparin was determined for maintaining ACT > 480 s with the Hemochron Jr. Signature+. Changes in ACT measured with different devices are shown below. ACT measured by the Hemochron 401 was not available after heparin administration because baseline ACT was markedly prolonged (308 s). n/a: not available

The ACT was 393 s after administration of heparin 14,000 U (300 U/kg), followed by an additional 2000 U before CPB. Heparin was added to maintain a steady concentration of 2.5–3.0 mg/kg and ACT > 480 s (Fig. 1). During CPB, the body temperature was gradually decreased to 30 °C. The aortic valve was replaced with a 23-mm Inspiris Resilia valve (Edwards Lifesciences, Irvine, CA, USA). The CPB and aortic cross-clamp times were 93 min and 72 min, respectively, and weaning from the CPB was uneventful. At the end of the CPB, an HPT test was performed. The heparin concentration was 2.5 mg/kg, and the protamine dose required to neutralize heparin on calculation was 120 mg. After protamine administration, the ACT returned to 121 s. Two units of fresh frozen plasma and two units of apheresis platelet concentrates were transfused intraoperatively.

The postoperative chest tube drainage volumes at 6- and 12-h post-surgery were 192 mL and 242 mL, respectively. The blood test results at the intensive care unit admission were as follows: platelet count, 97 × 10^9^/L; PT-INR (international normalized ratio), 1.16; APTT, 107.4 s; and plasma fibrinogen level, 261 mg/dL (reference range, 200–400 mg/dL). No additional blood transfusion was required. Eight hours after admission to the intensive care unit, an intravenous unfractionated heparin infusion was administered for postoperative anticoagulation treatment, and the APTT target was set as 150–200 s (1.5–2.0 times the baseline value). The patient was extubated on postoperative day 1, and oral warfarin therapy was initiated. There was no sign of organ failure suggesting catastrophic APS, and the patient was discharged on postoperative day 20 without any complications.

## Discussion

In the present case, we successfully utilized ACT monitoring using the Hemochron Jr. Signature+ for intraoperative anticoagulation management in patients with APS undergoing cardiac surgery, comparing its values with heparin concentration measured with the HMS Plus. No thrombotic or hemorrhagic complications were observed during the clinical course.

APS is an autoimmune disorder characterized by the presence of antiphospholipid antibodies, such as anti-β2GPI and anti-prothrombin antibodies [6]. The binding of these auto-antibodies to their antigen molecules activates clotting cascades, followed by platelet aggregation and thrombus formation. Contrary to the hypercoagulable nature of the syndrome in vivo, laboratory coagulation tests show spontaneously prolonged APTT and ACT since the interaction of antiphospholipid antibodies in patients’ plasma and phospholipids delays the clot formation in vitro.

Various approaches have been proposed for the intraoperative anticoagulation monitoring during cardiac surgery in patients with APS, but there is no clear consensus. The preferred technique in several reports is the measurement of whole blood heparin concentration during CPB with the HMS Plus [7, 8]. The advantages of the HMS Plus are as follows: (1) it can be used at the bedside for point-of-care testing, and (2) it can detect inadequate anticoagulation (heparin concentrations below than 3 U/mL during CPB) despite a prolonged ACT. However, the survey of members of the Society of Cardiovascular Anesthesiologists in 2017 demonstrated that only 13.1% of institutions answered that they used heparin concentration to determine adequate anticoagulation for CPB [9]. This result suggested that heparin concentration measurement devices such as the HMS Plus are not currently available in the majority of institutions providing cardiac surgery.

In contrast, the ACT measurement is universally accepted for routine monitoring during CPB. As mentioned above, the ACT values are often spontaneously prolonged in patients with APS; however, the ACT measured with the Hemochron Jr. Signature+ was not relatively affected in patients with APS as indicated in a previous report [5]. In the present case, the ACT over 480 s corresponded to the heparin concentration of 2.5 mg/kg (equivalent to 3.4 U/mL), which was adequate for anticoagulation during CPB. Prolongation of the ACT was not observed with the Hemochron Jr. Signature+, probably because its cartridge was preloaded with a sufficient dose of phospholipid, which could completely neutralize the effect of antiphospholipid antibodies. In contrast, the test tubes used in other devices (such as the Hemochron 401 and the Hemochron Response) only contained celite or kaolin [10].

Thus, for institutions where heparin concentration measurement devices are not available, the strategy of using ACT monitoring with the Hemochron Jr. Signature+ and maintenance of the ACT over 480 s for patients with APS during CPB may be recommended. The strength of the strategy is the simplicity and wide availability among institutions. However, this strategy has several limitations. First, the strategy is derived from the successful management of only one patient. Further examination of no excess ACT prolongation with the Hemochron Jr. Signature+ in patients with APS is needed for the generalization of our findings. Second, ACT is affected by various factors, such as hemodilution, hypothermia, and thrombocytopenia. Whether the ACT monitoring using the Hemochron Jr. Signature+ is adequate for every case (for example, aortic surgery with hypothermic circulatory arrest) in patients with APS is unclear. In addition, APS is known to have various clinical phenotypes. Thrombotic risk and antiphospholipid antibody activity are the highest in patients who are “triple-positive” (positive lupus anticoagulant, anticardiolipin, and anti-β2GPI antibodies) [11]. In the present case, only lupus anticoagulant was positive, and antiphospholipid antibody activity would not be so strong. We need to examine the utility of the Hemochron Jr. Signature in patients with “triple-positive” APS patients in whom the ACT values would be most affected.

## Data Availability

The data in this case report are available from the corresponding author on reasonable request.

## Abbreviations

APS: Antiphospholipid syndrome
APTT: Activated partial thromboplastin time
ACT: Activated clotting time
CPB: Cardiopulmonary bypass
HMS: Hemostasis Management System
PT: Prothrombin time
dRVTT: Dilute Russell’s viper venom time
HPT: Heparin protamine titration
INR: International normalized ratio
